# When artistic is altruistic: the *power of beauty* from P. A. Sorokin’s sociology to *Building Beauty* social project

**DOI:** 10.3389/fsoc.2024.1406156

**Published:** 2024-08-09

**Authors:** Licia Paglione

**Affiliations:** Department of Social and Political Sciences, Economics and Management, Istituto Universitario Sophia, Figline e Incisa Valdarno, Italy

**Keywords:** Sorokin, beauty, artistic action, love, creativity, fraternization, integral epistemology

## Abstract

The article contributes to the ongoing debates on the social value and sociological relevance of the arts by examining the intuitions of the Russian-American sociologist P. A. Sorokin (1889-1968) on the concept of “beauty” as a force akin to what he calls “Altruistic Creative Love”, both potentially catalysing a process of “fraternisation of humanity”. Starting from the author’s sociological reflections on the relationship between “Altruistic Love” and “beauty” and an analytical model of “altruistic artistic social action,” the article proposes the analysis of a specific social project named *Building Beauty*, promoted in Turin (Italy) by universities, public bodies and the third sector, which aims to foster the social inclusion of homeless people through participatory processes, discovering expressions of beauty with aesthetic and sociological relevance simultaneously, able to move social transformations.

## 1 Introduction: when the beauty matters (sociologically also)

In addition to the aesthetic value, beauty - understood in a general sense to include the various and varying concepts that refer to a certain harmony and proportion of forms and colours and their overall configuration (Morris, 1958, p. 314) - and in particular, action aiming at beauty – i.e. artistic action - could also contain a social and sociological value, influencing social transformation. As some sociologists suggest, this idea could indicate a direction of sociological research to discover beauty’s function in our society (Morris, 1958, p. 314). It is an intuition supported, for instance, by the Russian-American sociologist Pitirim A. Sorokin (1889–1968). In this article, his thought is analysed to highlight the sociological relevance of action aimed at beauty (i.e. artistic action) as a form of social action that, when combined with the Sorokinian “Altruistic Creative Love,” is potentially generative of a process of “fraternization” of the humanity (Sorokin, 2002, p. 479). Following this intuition, the article is structured in two parts. In the first theoretical part, building upon previous analysis (Paglione, 2022), Sorokin’s sociological reflection on “altruistic creative love” will be presented (1), and the link between this concept and the value of “beauty” will be clarified (2), and an analytical model of “altruistic artistic social action,” rooted in Sorokinian perspective, will be proposed, formed by five dimensions through which we can observe forms of beauty that are not only aesthetically, but also sociologically relevant (3). In the second empirical part, the article will present an empirical analysis, conducted in the light of these dimensions, of a specific social project - called *Building Beauty* - developed in Turin (Italy) in 2014 and promotes social inclusion of marginalised people through participatory processes creating of artefacts of both aesthetic and sociological value, highlight the social transformative “power of beauty,” i.e. the relevant sociological function of the arts also in the contemporary society (4). In this way, the article touches on the “uneasy relationship” between the arts and our society (Morris, 1958, p. 312) and suggests, in conclusion, the possibility of stimulating the integration or reintegration of fine arts into the contemporary human and social life and to recognise a role for the artists in social transformation.

## 2 Pitirim A. Sorokin: a sociology of creativity *embedded* in the *power of love*

Amongst the reflections on the social value and sociological relevance of beauty, some elements from the thought of Russian-American sociologist P. A. Sorokin (1889–1968) stand out (Federici, 2022; Paglione, 2022). In particular, in *The Ways and Power of Love* (2002 [1954]) - an ambitious book from the author’s most mature intellectual season entirely dedicated to an original analysis of the phenomenon of love - we can find intuitions about the sociological value of a type of love with an “altruistic” nature and great “creative” potential, closely connected to beauty. Although Sorokin came to study *Altruistic Creative Love* - as he defined the phenomenon - only in the latter part of his life, the roots of his interest are much earlier, linked to the author’s biographical origins (Ponomareva, 2011; Marletti, 2022) and his original cultural ‘journey’ (Sorokin, 1963) and matured within the framework of his more general sociological perspective which - later and amongst other things - was interpreted as a “sociology of creativity” (Sorgi, 1990, p. 15). The interest in love and its creative potential emerged in Sorokin as a reaction to the social “phase of disintegration” (Sorokin, 1956) - in line with the Durkheimian conception of the sociality as the source of the sacred and altruism as the basis of social solidarity (Jeffries, 2016; Mangone, 2020) that the society of his time, characterised by a “sensate cultural super-system” - dominated by values such as egoism, hedonism, utilitarianism - was going through (Sorokin, 1957). Amid bloody conflicts, struggles, and exasperated competitiveness, it was an “absolute necessity” - according to Sorokin - to find ways to study and implement a creative and unifying “energy” of love using sociological tools. According to Sorokin, the mission of sociology - a ‘committed’ science for him - was to help “understand the nature, forms, and how and why of love, but also to endeavour to design more efficient techniques for its production” (Sorokin, 2002, p. 37) in order to support a “programme of altruization” of people and institutions, necessary so that the overwhelming power of love could be unleashed and generate a “humanity united in one deeply united family” (Sorokin, 1955, p. 2). The scientific mission of Sorokin was not to be limited to producing theoretical descriptions of the qualities of love as a “moral and spiritual fact” but, following sociological epistemology, also required “historical, statistical, even biological investigations [...] experiments and research of a typically sociological nature into the concrete (individual and social) effects of altruistic love” (Sorgi, 1985, p. 155). At the same time, this task required an epistemological renovation of sociology (Mangone and Dolgov, 2019; Cipolla, 2022) oriented to overcome the dominant “quantum-phrenic” and “numerolatrous” approach that reduced sociology to a pure research technique, cut off from its depth of values and meanings. The sociological study of love required the development and adoption of an “integral” method of investigation - as Sorokin defined it - capable of grasping the multidimensional reality characterising the nature of social phenomena and the human being – a “marvellous integral being” (Sorokin, 1958, II) -, which is not only biological and rational, but also “super-sensorial and super-rational,” i.e., spiritual, intuitive, and creative. In the light of this integral approach, Sorokin conceptually defined Altruistic Creative Love as a multidimensional and dynamic phenomenon: “an inexhaustible universe in quality and quantity” that manifests itself in “many forms” (Sorokin, 2002, p. 3), at least seven, some visible, some not: religious, ethical, ontological, physical, biological, psychological and social. Sorokin focused on the latter two: the psychological form and the social form. In the first form, Love coincides with an “altruistic” by its very nature (Sorokin, 2002, p. 10) experience at the emotional, affective, volitional and intellectual levels at the same time (Sorokin, 2002, p. 9). The psychological form of love entails the identification of the one who loves with the one who is loved, without an annulment of the individuals: for the lovers, this experience coincides with “the loftiest form of freedom” (Sorokin, 2002, p. 11) and the expansion of “true individuality” (Sorokin, 2002, p. 10). In this sense, for Sorokin, love seems to be “creative” not only of social relationships but also of the human being, coinciding with the “highest peace of mind and happiness” (Sorokin, 2002, p. 12). In the second form, the social one, love is understood by Sorokin as “a meaningful interaction - or relationship - between two or more persons, where the aspirations and aims of one person are shared and helped in their realisation by other persons” (Sorokin, 2002, p. 13). In this relationship, the author identifies the “supreme and vital form of human relationship” (Sorokin, 2002, p. 76), one that allows the existence of the “happiest” human society and the realisation of the human being as an “integral being” because “it is to complete oneself in and through others” (Sorokin, 2002, p. 13). For the empirical observation of these two forms of love, which Sorokin jointly calls “psycho-social love,” the author proposes an analytical breakdown that encompasses some of their qualities to be considered dimensions of an analytical model with “theoretical and practical significance” (Sorokin, 2002, p. 19), useful for seeing the hidden part of an “iceberg” (Sorokin, 2002, p. 3), which is not well known and is underestimated by the contemporary “sensate culture.” According to the Sorokinian model, there are five dimensions of love (Sorokin, 2002, chap. II): intensity, which relates to the greatness and preciousness of what is freely given; extensivity, which varies from exclusive love for oneself to love for entire humanity, all living creatures and the entire universe; duration, which can vary from a brief moment, as may occur in a heroic act, to years or the entire course of a lifetime, as in a mother’s experience of caring for her child; purity, which relates to the logic and motivations that animate people and “varies from love that finds its *raison d’être* only in love itself” - without the taint of a “contaminating motivation” of utility, pleasure, advantage or profit - to “contaminated love,” as a means to utilitarian and hedonistic finalities; adequacy, which concerns the more or less congruent relationship between the subjective motivations of the act of love and its objective consequences. Following the Sorokinian proposal, according to these dimensions and the relations between them, it is possible empirically to discover the presence of creative love, animating a process of fraternization of humanity, or, on the contrary, a love “contaminated” by egoism, hedonism, utilitarianism, which is productive of coercive or contractual social relationships (Sorokin, 2002, p. 76), and indeed of social disintegration.

## 3 Beauty: more power for the altruistic and creative power of love (and *vice versa*)

Following Russian Orthodox culture and philosophy,^1^ which profoundly influenced Sorokin’s thought (Tiryakian, 1988; Gambescia, 2000; Abbottoni, 2004; Nichols, 2012), also in the American period (Ponomareva, 2011),^2^ Altruistic Creative Love is conceived as Goodness closely connected to - or better “integrated” with - two other great values: truth and beauty. In their integration, they form a “supreme value”:

“Among all the meaningful values of the superorganic world, there is the supreme integral value, the veritable *summum bonum*. It is the indivisible unity of Truth, Goodness, and Beauty” (Sorokin, 1958, IV).

“Love – Sorokin writes also – is viewed as goodness inseparable from truth and beauty. All three are unified aspects of the Absolute Value” (Sorokin, 2002, p. 6), and all three produce the “same effect,” contributing to the same creative function of individuals and society. In other words, Love shares the same integrative sociological function with Truth and Beauty: dynamically reuniting that which is separate (Sorokin, 2002, p. 47).

“If real beauty,”- Sorokin writes - “produces a catharsis in us, then real love or goodness impresses us as the purest and noblest beauty. It is no coincidence that the terms love and beauty are often used indifferently [...]” (Sorokin, 2002, p. 31).

Hence, the possibility that not only goodness (love), but also beauty could contribute to social transformation. According to Russian culture and especially the theological reflection of intuitivism, beauty represents a fundamental element in human and social life.^3^ As Abbottoni (2004, p. 32) remembers, the work that most influenced the spiritual education of the Russian people is the *Dobrotoliubje* or *Philokalia.* Sorokin, rooted in this culture, was born in a specific area populated by the Komi group, characterised by a deep sense of aesthetics. This is recognised by the scholar himself, who points out that the strong aesthetic culture of the Komi also embellished his existence, perceptibly shaping his tastes as an aesthete for the rest of his life (Sorokin, 1963, p. 16). Therefore, beauty is fundamental in Sorokin’s thinking and artists - defined as ‘heroes’ of beauty by the author - are considered social actors who can play an important sociological role. “All great and small creators in the field of […] real beauty” (Sorokin, 2002, p. 38) - Sorokin highlights - are to be counted amongst the “inventors and engineers of love production” (Sorokin, 2002, p. 38) and represent a “great power station, endlessly generating an enormous amount of the best love energy” (Sorokin, 2002, p. 40), a “creative energy that unifies, integrates and harmonises” (Sorokin, 2002, p. 6), one that is “indestructible” (Sorokin, 2002, p. 40) even beyond the lifetime of its producers, through “techniques of altruization” among which Sorokin explicitly mentions the “use of fine arts” (Sorokin, 2002, p. 317). The element that allows these ‘heroes’ to contribute to the creation of love resides in a specific mental structure and a well-developed dimension, which is fundamental to discovery and creativity in the artistic field: the “superconscious” (Sorokin, 2002, Chapter VI). In Sorokinian thought, this dimension represents a fourth level of personality or mental activity (in addition to the subconscious, the biological conscious, and the socio-cultural conscious) that “creates and discovers through *supraconscious intuition*” (Sorokin, 2002, p. 99). In this sense, Sorokin writes:

“All great creators in these fields have been persons graced by a generous magnitude of supraconscious, and this superconscious - in co-operation with the consciously learned skills and techniques - has been responsible for their achievements” (Sorokin, 2002, p. 105).

To clarify this aspect, Sorokin refers to creative processes experienced by exponents of the fine arts in which it is evident that the rational mind is insufficient for creativity (Sorokin, 2002, p. 107). Instead, the latter is nourished by a force, in which - and here Sorokin quotes Schelling - “the artist seems to stand under the influence of a power[...] which compels him to declare or represent things which he himself does not completely see through, and whose import is infinite” (Sorokin, 2002, p. 109). The two values, love and beauty, would thus seem to coincide in Sorokin’s thought. However, analysing more deeply what is written in *The Ways and Power of Love*, one realises that there is no complete overlap between them. On the one side, whatever is touched by love becomes beautiful (Sorokin, 2002, p. 78): love, therefore, creates beauty, represents “in a certain sense [...] its essential component” (Sorokin, 2002, p. 78), and in so doing, “increases its power” (Sorokin, 2002, p. 78). On the opposite side, authentic beauty, or rather action in the fine arts, is seen as a creative source of love: the fine arts “can and do serve the task of altruistic ennoblement” (Sorokin, 2002, p. 318). Love and beauty, then, for Sorokin, are two different values, mutually influencing each other in the form of “support” (Sorokin, 2002, p. 318) toward the same purpose. To emphasise this further, thinking of a sort of interchangeability between those who act in these fields - that is, between those dedicated to goodness (love) and those dedicated to the pursuit of beauty – Sorokin explains that “Their external garb changes, but their real function remains the same: they act as power stations generating the energy of love for humanity” (Sorokin, 2002, p. 40). This idea of a mutually supportive link between love and beauty seems to suggest that, although these values may also exist independently, their power to unify, integrate, and harmonise on a personal and social level - which is also sociologically relevant - depends on the degree to which they are integrated: beauty could be all the more creative, the more it is nourished by love. This position is further clarified by the Sorokinian idea that the three values of truth, goodness and beauty, because all part of the same Ultimate Reality, can transform into each other:

“[…] since real truth, real beauty, and real goodness or love are three inseparable aspects of the Ultimate Reality, the possibility of the transformation of one of these ‘energies’ into the other two follows logically from the hypothesis [...]. *Real beauty*, whether in the form of great music, great literature, drama, painting, sculpture, or architecture, *simultaneously purifies and ennobles us morally*” (Sorokin, 2002, p. 30).

The possibility of transforming one of these energies into the others means, on the level of social action, that “all those who enriched humanity with truth and beauty have also contributed to a more efficient production of love” (Sorokin, 2002, p. 39). Therefore, it means that an artist or a seeker of beauty can contribute with their actions to create love and truth. However, the transformation of truth-goodness-beauty into each other - Sorokin emphasises - takes place partially as it “always gives an efficiency below 100 per cent” (Sorokin, 2002, p. 31) due to the influence of certain factors linked to the “qualitative-quantitative magnitude of each energy” (Sorokin, 2002, p.31). Sorokin sees these factors grouped around the five dimensions proposed for the analysis of love:

“The intenser, the purer, more extensive, durable, and adequate the given energy of truth or beauty is, the greater tends to be the percentage of its efficient transformation into goodness (love) energy; and vice versa” (Sorokin, 2002, pp. 31–32).

Sorokin further emphasises this aspect in his chapter on the techniques of altruistic transformation (Sorokin, 2002, Chapter XVII), including the fine arts. Here, recalling the connection between the values of beauty, truth and goodness, he again makes it explicit that artistic activity can play a relevant role with regard to the altruization of people:

“All the genuine fine arts have been serving and can increasingly serve the tasks of the spiritual and moral ennoblement of the man” (Sorokin, 2002, p. 319).

The terms *genuine* or *real* qualifying the fine arts are used by the Russian-American sociologist to mark a differentiation between them and the “vulgar pseudo-arts” (Sorokin, 2002, p. 318), the second being ineffective and unable to “radiate the light of the highest values” (Sorokin, 2002, p. 320) and which were unfortunately predominant in his times:

“[…] the bulk of the modern arts is overwhelmingly negativistic, muckraking and “dirt-painting.” […] The high-brow modern arts are mediocre in their artfulness, whilst the modern popular art are conspicuously vulgar” (Sorokin, 2002, p. 320).

Pseudo-arts risk, in contrast to “genuine fine arts,” having “demoralising, disintegrating, and enervating effects rather than any constructive influence” (Sorokin, 2002, p. 320) by being useless for the realisation of altruistic transformation and the expression of the creative power of society and human beings. These elements highlight Sorokin’s intuition that even beauty and its creators (artists) can, by acting artistically, contribute sociologically to creating and recreating social integration, not automatically, but to the extent they are nourished and unified by the “indispensable component” (Sorokin, 2002, p. 78) of altruistic love. In turn, they become generative of the latter, feeding a virtuous circle in the production of what to Sorokin seems to be an energy of absolute necessity for the harmonious existence of society.

## 4 Toward beauty as a form of love and a definition of altruistic artistic social action

In Sorokin’s thought, therefore, beauty as an expression, on the one hand, and origin, on the other, of that energy of Altruistic Creative Love, contributes to the programme of altruization of humanity, that is, it can perform the same creative function as love. Indeed, beauty could be understood, after all, as a further form of altruistic creative love, in addition to the seven forms explicitly identified by Sorokin. Along this line of thought, but going further than Sorokin’s proposal, it might be sociologically interesting to observe beauty at the empirical level and thus develop analytical models of *creative artistic social action* with an altruistic significance and the typical effects of love in order to enable the “fraternization of humanity.” Sorokin himself would seem to offer some useful suggestions for formulating such a model. A first element resides in emphasising the necessary unity between beauty and the two other values of the Supreme Value - goodness and truth - in types of social action that Sorokin calls “objectively altruistic,” i.e. having real fraternising effects.

“[…] these *objectively altruistic actions are possible mainly through the indivisible unity of goodness, truth, and beauty, and the possibility of their mutual transformation into one another*. If, in their ontological nature, they are inseparable - though distinct in their individuality - anyone who truly creates in one of these fields indirectly creates in the other two: genuine goodness is always true and beautiful; genuine beauty is always good and true, and genuine truth is always good and beautiful. The great and small creators in the fields of truth and beauty may not be directly motivated by goodness (love); yet when they are creative in these fields, they cannot help becoming creative also in the field of love because of the unity and mutual transformability of these ‘forms of energies’” (Sorokin, 2002, pp. 18–19).

From the Sorokinian perspective, it would seem that social action is capable of realising “genuine beauty” in the artistic field and, at the same time, is capable of contributing to increasing goodness and truth. The unity of the three values (beauty, goodness, and truth) is necessary for artistic actions to influence social life *objectively*. A second related element explains this possibility of influence according to different gradations, introducing reflection on the degree of efficiency with which these forms of energy can transform one into the other. This intuition opens the way to examining whether, how, and how much the beauty-seeking action is simultaneously creative of goodness (love) and thereby contributes to the integrative sociological function of society. This aspect, building upon Sorokin’s thought, would express a measure of the authenticity of beauty, resulting from its being unitedly integrated with other values. In attempting to translate this idea empirically, useful insights can be found in the five dimensions that Sorokin proposes to observe Altruistic Love empirically: purity, intensity, adequacy, extensity, and duration. Following Sorokin’s thought, these dimensions can constitute a model for analysing beauty in its capacity to create elements essential for social integration (Sorokin, 2002, p. 32). By way of example, Sorokin applies this model to the analysis of the great compositions of Bach, Mozart and Beethoven, emphasising, in particular, the importance of duration through time: “great art creations endure and continue to be transformed into goodness for generations, centuries, millennia” (Sorokin, 2002, p. 32). Through these elements, one could attempt to propose an analytical model of “altruistic artistic action” for observing actions in the artistic field concurrent with actions related to the sphere of goodness and truth in the process of fraternization, measuring their efficiency in a sociological sense. Table 1 describes this analytical model and its operational dimensions: purity, intensity, extensity, adequacy, and duration.

**Table 1:** Analytical model of Altruistic Artistic Social Action.

Altruistic Artistic Social Action		
Empirical dimensions	Minimum	Maximum
Intensity	Zero interest	Infinite interest
Purity	Only utilitarian motivation	Only love as motivation
Extensity	Oneself only	Whole universe
Duration	Short moment	Whole life
Adequacy	Discrepancy between subjective goals/objective consequences	Identity between subjective goals/objective consequences

The graduation of the scale of each dimension, inspired by the Sorokinian perspective and words, is purposely generic so that it can be adapted according to the different phenomena to be analysed. However, on the whole, it would like to represent the basis for making possible the observation of an artistic social action capable of contributing to an altruistic transformation of humanity. Depending on the characteristics of these dimensions, the artistic actions could result more or less capable of expressing the “power of love,” thereby contributing to altruistic social transformation, which makes individuals and groups become “more altruistic and creative beings who feel, think and act as true members of a humanity united in one profoundly supportive family” (Sorokin, 1955, p. 2). This model, appropriately adapted, could be used as a specific analytical tool to observe artistic phenomena, in the hypothesis that it contributes to creating *places* not only for the artist’s expression or the entertainment of the ‘inhabitant’ but also for the creation of “supreme and vital forms of human relations” (Sorokin, 2002, p. 76), thus making beauty or artistic action container of aesthetic and, at the same time, sociological values. Following this hypothesis, the next section proposes an analysis of a specific social project based on artistic activities - called *Building Beauty* - aiming to grasp its sociological value.

## 5 *Building Beauty*: fraternizing art in (social) action

Beyond the idea of the independence of arts and artists from society, shared by traditional aesthetic visions, and beyond the deterministic sociological point of view of arts as a manifestation of socio-cultural-economic circumstances, the article conceives arts as a force influencing the culture and society: a concrete and public activity oriented to produce, at the same time, beauty and social transformation. In this sense, the analysed social project - *Building Beauty* - aims to operate:

“Beauty is understood as a concrete experience, which we can all build and enjoy so that we have one more tool to act in the world” (Porcellana, 2019).

Through these words from the anthropologist Valentina Porcella, one of the ‘parents’ of the project Building Beauty, with the designer Cristian Campagnaro, and also through the project’s name, it is easy to understand the central place occupied by the value of beauty and the role of artistic creative action. Using their recent books as a “life story” of the project and other documents (scientific articles, newspaper articles and video) and analysing their content in the light of Sorokin’s analytical perspective, in particular, through the previously proposed definition of “altruistic artistic social action,” it becomes possible to understand whether and to what extent the pursuit of beauty and the use of art creative on a sociological level, promoting social integration or, in Sorokian words, “fraternization.” Launched in July 2014 - and based on the previous project *Abitare il dormitorio*^4^ – in the northern suburb of Turin (Italy), in a building made available by the municipal administration, the project was created as part of the collaboration between the Dept. of Architecture and Design of the Polytechnic University of Turin, the Dept. of Philosophy and Educational Sciences of the University of Turin, the Service for the Prevention of Social Fragility and Support for Adults in Difficulty of the City of Turin and the Cooperativa Animazione Valdocco Onlus, on the theme of combating homelessness (Campagnaro and Ceraolo, 2022, p. 178). *Building Beauty* consists of a permanent creative workshop, active twice a week, to promote the social inclusion of marginalised people (mostly *homeless*). The project was born in a space formerly used as a shelter for people “with a health situation seriously compromised by years of living on the streets, suffering from various illnesses and awaiting special declarations of invalidity” (Porcellana, 2019, p. 54), providing them with a “short-term reception” (Porcellana, 2019, p. 57). On these premises, and through an observational intervention by anthropologists and designers, the shelter began to be thought of differently, involving the collaboration of various local bodies, including the university (Porcellana, 2019, p. 59), and of the shelter guests themselves in creative activities, initially using material found available in municipal warehouses, particularly chairs, but not only. As Campagnaro and Ceraolo (2022, p. 180) write:

“Over the years, *Building Beauty*’s workshops have produced all kinds of ‘things’: chairs, armchairs, bookcases, shelves, tables, lamps, partitions, benches, outdoor furniture, children’s toys, displays, desks, jewellery, clothes, hats, aprons, wall painting, wayfinding systems, renovated domestic interiors, gardens and courtyards reclaimed from abandonment, lunches, lunch boxes, neighbourhood parties, exhibitions, and manual workshops open to the public.”

Beyond the artistic handcrafts, the overall product of *Building Beauty* is more general and concerns a “change in perspective.” As Porcellana (2019, p. 67) writes:

“Starting with the tailoring, woodworking and cooking workshops, the new project [*Building Beauty*] would not be a single extraordinary event but would propose a series of parallel workshops taking place twice a week [...]. The change in perspective had displaced us first: no longer a short and intended course to spend our professional skills and collect experience, but a daily and prolonged immersion in a reality to be invented.”

Campagnaro and Ceraolo (2022, p. 180) highlight the unrepeatability of the products as a result of unique conditions:

“What we produce in *Building Beauty* is unique and unrepeatable; it is so because the conditions that contribute to the success of the project are variable and depend on who participates, the resources available, the skills and practical abilities of each person present, as well as their desires and visions and, why not, the moods and emotions of the moment. These are forces that shape those “things” and influence the processes. The latter, on the other hand, develop according to a well-established and constant process: open to experimentation and error, they have as their main driver the creative and manual collaboration between the participants and are organised in such a way as to promote everyone’s self-efficacy and interpersonal skills, using them as a valuable resource.”

On this basis, *Building Beauty* seems to be a form of action motivated predominantly by “purity,” in Sorokin’s terms, i.e. not egoistic motivations, intense and tending toward a prolonged duration over time. The *Building Beauty* workshops attracted not only various organisations gradually but also homeless people outside the shelter, expanding over the years what Sorokin would also call the “extensity” of creative action. Porcellana tells:

“Some of the women trainees were guests at the facility, whilst others, men and women, were housed in dormitories or reception centres [...] in even distant parts of the city; they had got up early to reach the workshop by public transport” (Porcellana, 2019, p. 68).

The effect of this type of creative action, evident in the community of practise that is *Building Beauty*, is linked to a power, as Porcellana (2019, p. 41) calls it:

“Within the community of practise, we all experience an increase in power, not of an individual and competitive kind, but of a collaborative nature.”

Such power is strongly reminiscent of what Sorokin attributed to love, especially in its social form, as the “supreme and vital human relationship” (Sorokin, 2002, p. 76). The type of relationship experienced in the workshops is based on “names and surnames,” “recognising the full dignity of people, in an atmosphere of attention and care that diluted the bureaucratic nature of the Social Services” (Porcellana, 2019, p. 71) and recalling the idea of the “wholeness” that the person experiences in Sorokin’s relationships of love (Sorokin, 2002, p. 13). In this sense, it would seem that the action present in *Building Beauty* also has a good degree of Sorokinian “adequacy,” producing in the participants an awareness of their own value and abilities and a deep sense of belonging:

“When I leave here, I do not think: I’m done, goodbye and thank you, and no more about it. When people ask me what I do, I always say: ‘Building beauty’! (B., one of the workshop participants, in Porcellana, 2019, p. 72).

Such a relationship is built together and through the pursuit of beauty, recalling the Sorokinian idea of mutual “support” between goodness and beauty, which is expressed here through the creative artistic production of objects and, with them, of “special bonds” (Porcellana, 2019, p. 71):

“Everything in our case is focused on people and the relationship between people. The handicrafts made and the tools used in daily practise are there to create opportunities for dialogue and understanding between participants” (Porcellana, 2019, p. 37).

In the words of Porcellana (2019, p. 20), this effect can be grasped with great awareness:

“We were all surprised, designers included, at the unexpected forms that objects co-created by so different people in terms of life experience, age, social background, and origin could take. The handicrafts [...] have been a powerful tool for connecting with an ever-widening network of actors who have related to the project by capturing [their] beauty [...].”

From this analysis, according to the Sorokinian perspective, it is possible to capture the solidaristic effect of beauty as the result of a particular type of artistic action endowed with specific qualities and quantities of each of the five dimensions of altruistic love. The analysis of *Building Beauty* shows strong intensity and high purity in motivation, great extensiveness, long duration and adequacy of the social action of love. The “beautiful” artefacts in *Building Beauty* thus seem to perform a double creative function: aesthetically and sociologically, as they express the “power” of integrating everyone, promoting a fraternization process, starting with marginalised people, into a community (of practise) that resembles what Sorokin would call “united humanity” in a “profoundly supportive family” (Sorokin, 1955, p. 2).

## 6 Conclusion

The Sorokin’s thought offers a critical perspective to obverse the sociologically relevance of beauty - or, better, artistic social action, as a form of act aiming at beauty - also in the contemporary society. In particular, the concept of “altruistic artistic social action,” rooted in Sorokin’s thought, and its use in empirical analysis could represent a contribution to the debate concerning the sociological value of beauty and its influence of arts on social transformation. Beyond the analysed phenomenon, the Sorokinian perspective can also highlight cases of failure of artistic actions in creating solidarity. Fraternization as a result of the artistic action - i.e. the possibility of arts producing an integrative sociological function - is not obvious: it depends, according to Sorokin, on the contamination of artistic action with that “indispensable component” (Sorokin, 2002, p. 78) which is Altruistic Love. Moreover, since the five proposed analytical dimensions intertwine in different ways, the artistic actions could differ in the quantity of each dimension and assume different possible nuances but obtain some results in solidaristic terms. In his analysis, Sorokin describes and distinguishes forms of arts - “pseudo-arts” in his words – as prevailing in his contemporary cultural situation or, in Sorokinian terms, “cultural super-system” - that are not or little contaminated by love. Using the Sorokinian perspective, therefore, it is possible to take a critical view of arts to discover not only the altruistic artistic action that is effective in the fraternization process but also artistic actions that are unable or less able to contribute in this direction. In this way, the Sorokinian perspective nuances the dichotomy, translating it into different possibilities that are more or less transformative of the society. The Sorokinian intuition regarding the connection between love and beauty offers, moreover, a critical interpretative key, challenging the traditional opposition between aesthetic and sociological disciplines (Wolff, 2021, p. 27) and unifying them in a global analytical perspective, coherent with the Sorokinian integral epistemology (Cipolla, 2022; Galluzzi and Paglione, 2024). In this sense, the Sorokinian intuition could open up various “pathways” for research, including interdisciplinary ones, on the revolutionary or prophetic role that artistic actions can play in society insofar as it remains anchored to a deep and mysterious dimension of the human being: the altruistic creative energy of love. In this direction, Sorokin’s intellectual heritage could contribute to imagining - in line with the figure of this author as a “sociologist of possibility” (Abbottoni, 2004, p. 104) - an original sociology of arts and innovative analytical tools - beyond the aesthetic frontiers and beyond the dichotomy between autonomy/determinism in the relationship arts/society - to capture the sociological value of artistic actions in social transformation and the “critical potential of people” (Cataldi and Iorio, 2023) - as artists - in fraternization process towards the “reconstruction of humanity” (Sorokin, 1948).

## Data availability statement

The original contributions presented in the study are included in the article/supplementary material, further inquiries can be directed to the corresponding author.

## Author contributions

LP: Conceptualization, Formal analysis, Investigation, Methodology, Writing – original draft, Writing – review & editing.

## Footnotes

^1^Many authors highlight the influence of Russian culture on Sorokin’s thinking. Some authors compare Sorokin’s reflections to Dostoevskij’s thought (Tiryakian, 1988), others to Tolstoy’s universalistic pacifism (Gambescia, 2000), and some describe him with the idea of a “Russian soul” (Nichols, 2012). A wide-ranging comparison between Sorokin and Russian philosophers and theologians, through which the roots of the integrated vision between the three values mentioned, is presented in Abbottoni, 2004. This author also describes the theological roots of the topic of beauty (Abbottoni, 2004, pp. 32–37).

^2^In this sense, see, for instance, Ponomareva (2011). The article compares the two periods of Pitirim A Sorokin’s life, career, and scientific work: the Russian period (until 1922) and the American period (1923–1968), highlighting that the first period is a prototype of the American one and not its polar opposite. Therefore, the concepts developed in the American period - Creative Altruistic Love also - are the development of his ideas emerging in the past in Russia.

^3^One of the most famous Russian writers, F. Dostoevskij (2014 [1973]), emphasised this cultural aspect in one of his novels: “Only beauty is indispensable because without it nothing could exist in the world!”

^4^*Abitare il dormitorio* was an action research set up in 2009 and led by the anthropologist Valentina Porcellana from the Department of Philosophy and Educational Sciences of the University of Turin and by the architect and designer Cristian Campagnaro from the Department of Architecture and Design of the Polytechnic of Turin. The project was dedicated to the social inclusion of vulnerable and marginalised people through the development of product and process strategies to strengthen the quality of temporary housing and new stable and durable housing forms (Campagnaro and Porcellana, 2016, p. 218).
