# Corrigendum: When artistic is altruistic: the *power of beauty* from P. A. Sorokin's sociology to *Building Beauty* social project

**DOI:** 10.3389/fsoc.2024.1493733

**Published:** 2024-10-11

**Authors:** Licia Paglione

**Affiliations:** Department of Social and Political Sciences, Economics and Management, Istituto Universitario Sophia, Figline e Incisa Valdarno, Italy

**Keywords:** Sorokin, beauty, artistic action, love, creativity, fraternization, integral epistemology

In the published article, there was a typographical error. A correction has been made to the Section **Pitirim A. Sorokin: a sociology of creativity** ***embedded*** **in the** ***power of*** ***love***.

This sentence previously stated:

“The interest in love and its creative potential emerged in Sorokin as a reaction to the social “phase of disintegration” (Sorokin, 1956) - in line with the Durkheimian conception of the sociality as the source of the sacred and altruism as the basis of social solidarity (Jeffries, 2016; Mangone, 2020) that the society of his time, characterised by a “sensualist cultural super-system” - dominated by values such as egoism, hedonism, utilitarianism - was going through (Sorokin, 1957).”

The corrected sentence appears below:

“The interest in love and its creative potential emerged in Sorokin as a reaction to the social “phase of disintegration” (Sorokin, 1956) - in line with the Durkheimian conception of the sociality as the source of the sacred and altruism as the basis of social solidarity (Jeffries, 2016; Mangone, 2020) that the society of his time, characterised by a “sensate cultural super-system” - dominated by values such as egoism, hedonism, utilitarianism - was going through (Sorokin, 1957).”

In the published article, there was an typographical error. A correction has been made to the Section **Pitirim A. Sorokin: a sociology of creativity** ***embedded*** **in the** ***power of*** ***love***.

This sentence previously stated:

“For the empirical observation of these two forms of love, which Sorokin jointly calls “psycho-social love,” the author proposes an analytical breakdown that encompasses some of their qualities to be considered dimensions of an analytical model with “theoretical and practical significance” (Sorokin, 2002, p. 19), useful for seeing the hidden part of an “iceberg” (Sorokin, 2002, p. 3), which is not well known and is underestimated by the contemporary “sensualist culture.””

The corrected sentence appears below:

“For the empirical observation of these two forms of love, which Sorokin jointly calls “psycho-social love,” the author proposes an analytical breakdown that encompasses some of their qualities to be considered dimensions of an analytical model with “theoretical and practical significance” (Sorokin, 2002, p. 19), useful for seeing the hidden part of an “iceberg” (Sorokin, 2002, p. 3), which is not well known and is underestimated by the contemporary “sensate culture.””

The authors apologize for this errors and state that this does not change the scientific conclusions of the article in any way. The original article has been updated.

